# Tiered healthcare in South Africa exposes deficiencies in management and more patients with infectious etiology of primary adrenal insufficiency

**DOI:** 10.1371/journal.pone.0241845

**Published:** 2020-11-05

**Authors:** Thabiso Rafaki Petrus Mofokeng, Kwazi Celani Zwakele Ndlovu, Salem A. Beshyah, Ian L. Ross

**Affiliations:** 1 Division of Endocrinology, Department of Medicine (University of the Free State), Free State, South Africa; 2 Division of Nephrology and Hypertension, Department of Medicine (University of Cape Town), Cape Town, South Africa; 3 Dubai Medical College, Dubai, United Arab Emirates; 4 Department of Endocrinology, Mediclinic Airport Road Hospital, Abu Dhabi, United Arab Emirates; 5 Division of Endocrinology, Department of Medicine (University of Cape Town), Cape Town, South Africa; Sahlgrenska University Hospital, SWEDEN

## Abstract

**Objective:**

We wished to determine the prevalence, etiology, presentation, and available management strategies for primary adrenal insufficiency (PAI) in South Africa (SA), hypothesizing a prevalence greater than the described 3.1 per million. There is great inequity in healthcare allocation, as two parallel healthcare systems exist, potentially modifying PAI patients’ clinical profiles, private being better resourced than public healthcare.

**Methods:**

An online survey of physicians’ experience relating to PAI.

**Results:**

The physicians were managing 811 patients, equal to a prevalence of 14.2 per million. Likely causes of PAI in public/ academic vs private settings included: AIDS-related [304 (44.8%) vs 5 (3.8%); *p*<0.001], tuberculosis [288 (42.5%) vs 8 (6.0%); *p*<0.001], autoimmune disease [50 (7.4%) vs 88 (66.2%); *p*<0.001], malignancy [27 (4.0%) vs 7 (5.3%); *p* = 0.500], genetic including adrenoleukodystrophy (ALD) [5 (0.7%) vs 16 (12.0%); *p*<0.001], respectively. Overall, more patients presented with nausea [101 (74.3%) and vomiting 89 (65.9%), than diarrhoea 76 (58.9%); *p* = 0.008 and 126 (15.5%) in adrenal crisis. Features suggestive of a crisis were hypoglycaemia [40 (78.4%) vs 42 (48.8%); *p* = 0.001], shock [36 (67.9%) vs 31(36.9%); *p*<0.001], and loss of consciousness [25 (52.1%) vs 27 (32.9%); *p* = 0.031]. Greater unavailability of antibody testing in the public vs. the private sector [32 (66.7%) vs 30 (32.1%); *p* = 0.001], [serum-ACTH 25 (52.1%) vs 16 (19.5%); *p*<0.001] and glucocorticoids were [26 (54.2%) vs 33 (40.2%); *p* = 0.015]. Many patients, 389(66.7%) were not using identification, indicating that they need steroids in an emergency.

**Conclusion:**

A survey of South African physicians suggests a higher prevalence than previously reported. Patients presented with typical symptoms, and 15.5% presented in adrenal crisis. Significant disparities in the availability of physicians’ expertise, diagnostic resources, and management options were noted in the public versus private settings. Greater awareness among health practitioners to timeously diagnose PAI is required to prevent a life-threatening outcome.

## Introduction

We previously identified the significant barriers to diagnosis and management of primary adrenal insufficiency (PAI) in Africa and that the etiology was predominantly related to tuberculosis (TB) and human immune deficiency virus (HIV) [1]. A single case finding study by Ross et al., [2] suggested a prevalence of 3.1 per million in South Africa (SA), but was limited by ascertainment bias. By contrast, overall, the prevalence in Western countries is 136 per million and 144 per million in European countries, respectively [3–5]. The underlying etiology has evolved from predominantly TB to autoimmune, especially in populations of European descent [6,7]. TB is a leading cause of death in Africa, and in 2016 was the leading cause of death in SA [8].

In a descriptive study of acute PAI in SA in 1999, before the peak incidence of Acquired Immune Deficiency Syndrome (AIDS) in SA, the underlying etiology was considered idiopathic in 42%, associated with active TB (18%), previous TB (16%), autoimmune (12%) and metastases in 6% [9]. Despite the high prevalence of TB in SA, autoimmune etiology accounted for 50% of a South African cohort [10]. However, the majority were of European descent, corroborating that autoimmunity predominated as SA’s underlying cause. Human immunodeficiency virus (HIV) is highly prevalent in SA, with 7 million infected persons, of whom more than 3.4 million are treated with highly active antiretroviral therapy [11]. Opportunistic infections (OIs) in AIDS have been implicated in PAI development [12–14], yet the real impact of these OIs causing PAI has never been assessed in any large study. Given the high prevalence of AIDS, there is likely a higher proportion of PAI patients attributable to HIV infection than previously thought.

South Africa has a complicated social and political history, with a long-standing inequitable resource allocation to its healthcare needs. The public healthcare sector, with an annual per capita expenditure of $ 131.73 caters to 37.9 million underprivileged people and, by contrast, the private healthcare with an annual per capita expenditure of $ 937(1) in (2005) allocated to 6.9 million persons of greater affluence [15].

To expand our understanding of PAI, we wished to survey medical practitioners’ perceptions, acknowledging that this methodology may have weaknesses. We also wished to understand how dichotomized healthcare systems may influence the clinical profiles of these patients. We hypothesized that a survey of medical practitioners in SA might reveal additional information relative to PAI prevalence and especially that tuberculosis and HIV related PAI may be more common than previously suggested.

## Materials and methods

### Design

This is a subgroup analysis of a previously published cross-sectional survey-based study [1]. For the creation, dissemination, and analysis of the questionnaire, Survey Monkey (SVMK Inc., San Mateo, California, USA) was used. A commercial database of clinicians, MedPages^®^, Green Point Cape Town, SA, was used to send e-mail invitations to medical practitioners, to confirm their willingness to participate in an online survey. The original questionnaire, and possible responses are available as an S1 Appendix. Briefly, the 23-questions covered several domains, including patient demographics, etiology, presentation, therapy, and barriers to diagnosis and treatment [1]. This report focuses on the sub-analysis of the responses of clinicians from SA to examine specific questions related to tiered service provision from a more homogeneous background. We have elected not to impute for missing data because of the concern that it may ignore the variability of the data and overlooks the relationship with other data. Although we encountered missing data, the median response rate was 83% (IQR, 81.4%-98.3%) for the public sector and private sector 81.6% (IQR, 78.6%-98.0%); *p* = 0.085. We have, therefore, only taken into account the number of responses out of the total responses.

### Ethics

The study was conducted in accordance with the ethical principles of the Declaration of Helsinki. Ethical approval was granted by the Faculty of Health Sciences Human Research Ethics Committee, the University of Cape Town, Republic of South Africa [Approval reference HREC/REF: RO42/2016]. Informed electronic consent was obtained from all participants before they could proceed to participate in the study. Data were retrieved and exported for analysis anonymously.

### Data management

Two types of data were captured. First, data describing the attributes of the respondents were retrieved directly from responses. Secondly, data characterizing patients were synthesized based on the numbers of patients seen by a given physician and the relative proportions of the physicians’ responses. However, no direct patients’ data were collected.

### Statistical analysis

Categorical and ordinal variables were summarised using proportions and percentages. Proportions and categorical variables were compared using Pearson’s Chi-square test or Fisher’s exact test, as appropriate. The responses were expressed in numbers and adjusted for any missing responses by expression as a percentage. Patient numbers were listed as declared by respondents for each patient inquiry with proportions calculated out of overall patient numbers declared for PAI. The combined total for each variable in the two groups (public/academic vs. private) was used to calculate the overall proportions, and associated confidence intervals were calculated using a logit transform. A two-sample test for proportions was used to compare the differences between overall proportions. A 2-sided *p*-value < 0.05 was considered as significant. All analyses were performed using STATA statistical software, version 15 (Stata Corp, College Station, Texas, USA).

## Results

### Demographic and professional profiles of participating physicians

Personalized e-mails were sent to 11 741 general practitioners, and 9 580 specialists, and a total number of 704 responses were received, of whom 303 completed the questionnaires. Of the completed questionnaires, 162 respondents confirmed that they treat patients with hypoadrenalism. Most of the respondents in both public vs. private were not endocrinologists [78/162 (48.2%) vs 12/162 (7.4%); *p*<0.001] and [37/59 (62.7%) vs 41/103 (39.8%); *p* = 0.005] identified themselves as non-endocrine specialists, [16/59 (27.1%) vs 56/103 (54.4%); *p* = 0.001] as general practitioners, and the remainder were endocrinologists [6/59 (10.2%) vs 6/103 (5.8%); *p* = 0.357], representing the minority, respectively. Across the entire cohort, the majority of clinician self-reported their grade as senior 104/155 (67.1%), compared with 9/155 (5.8%) who were junior doctors; *p*<0.001 (Table 1).

**Table 1:** Professional, and practice profiles of the participating physicians in the whole study group and by practice type.

Physician and practice setting	Public and Academic	Private	*P-* value^a^	Total (%, 95% CI)	*P-* value^b^
**Practice type**					
Private	-	103 (100)	-	103 (63.6, 55.7–71.0)	Reference
Academic	13 (22.0)	-	-	13 (8.0, 4.3–13.3)	**<0.001**
Public	46 (78.0)	-	-	46 (28.4, 21.6–36.0)	**<0.001**
**Career grades**					
Senior	37/58 (63.8)	67/97 (69.1)	0.498	104/155 (67.1, 59.3–74.1)	Reference
Middle grade	16/58 (27.1)	26/97 (26.8)	0.916	42/155 (27.1, 20.6–34.7)	**<0.001**
Junior (Trainees)	5/58 (8.6)	4/97 (4.1)	0.296	9/155 (5.8, 3.0–10.8)	**<0.001**
**Specialty**					
Non-endocrine specialist	37/59 (62.7)	41/103 (39.8)	**0.005**	78/162 (48.2, 40.2–56.1)	Reference
General practitioner	16/59 (27.1)	56/103 (54.4)	**0.001**	72/162 (44.4, 36.6–52.4)	0.504
Endocrinologist	6/59 (10.2)	6/103 (5.8)	0.357	12/162 (7.4, 4.2–12.6)	**<0.001**
**Practice locality**					
Urban	51/59 (86.4)	85/103 (82.5)	0.513	136/162 (84.0, 77.4–89.2)	Reference
Rural	8/59 (13.6)	18/103 (17.5)		26/162 (16.0, 10.8–22.6)	**<0.001**

Data are presented as frequencies and percentage = N(%).

CI, Confidence interval.

^a^*P*-value: Comparison between Public and Private sectors;

^b^*P*-value: Comparison across the entire group of medical practitioners who participated in the survey

n/N(%) = number of responses out of the total number of answers received.

### Characteristics of the patients, etiology, and presentations of patients

The data on 366 patients with pituitary disease, 426 patients with long-term steroid use, and 209 patients who underwent bilateral adrenalectomy were excluded from the analysis. The physicians’ responses were based on their own clinical, but contemporaneous experience of 811 patients, 459 were females and 352 males. The majority of patients, 713 (87.9%) were between 16–60 years of age, and 5.9% were 0–15 and 6.2% older than 60yrs.

The likely causes of PAI, based on the clinicians’ assessments, stratified for public vs. private health-care settings included autoimmune disease [50/678 (7.4%) vs 88/133 (66.2%); *p*<0.001], tuberculosis [288/678 (42.5%) vs 8/133 (6.0%); *p*<0.001], AIDS-related [304/678 (44.8%) vs 5/133 (3.8%); *p*<0.001], malignancy [27/678 (4.0%) vs 7/133 (5.3%); *p* = 0.500], genetic including adrenoleukodystrophy (ALD) [5/678 (0.7%) vs 16/133 (12.0%); *p*<0.001] and other [4/678 (0.6%) vs 9/133 (6.8%); *p*<0.001], respectively (Table 2). Among those considered to be autoimmune, additional autoimmune associations in descending order, included primary hypothyroidism [64/192 (33.3%) vs 81/190 (42.6%); *p*<0.061]), type 1 diabetes mellitus [60/192 (31.2%) vs 51/190 (26.8%); *p*<0.343], pernicious anaemia [35/192 (18.2%) vs 25/190 (13.2%); *p* = 0.173), premature ovarian insufficiency [20/192 (10.4%) vs 24/190 (12.6%); *p*<0.498] and Graves’ disease [13/192 (6.8%) vs 9/190 (4.7%); *p* = 0.394].

**Table 2:** Reported sex distribution, age groups and aetiology of the patients with adrenal insufficiency on which physicians based the whole study group and by practice type.

Demographics and clinical characteristics	Public and Academic	Private	*P*-value^a^	Total (%, 95% CI)	*P-*value^b^
**Number**	570	241	-	811	-
Primary adrenal insufficiency*	570/811 (70.2%)	241/811 (29.7%)	**<0.001**	811/1812 (44.8, 42.4–47.1)	
**Female sex**:	318/570 (55.8%)	141/241 (58.5%)	0.476	459/811 (56.6, 48.2–52.4)	
**Age groups**:					
0–15 years	34/570 (6.0)	14/241 (5.8)	**0.001**	48/811 (5.9, 4.3–7.8)	**<0.001**
16–60 years	513/570 (90.0)	200/241 (83.0)		713/811 (87.9, 85.5–90.1)	Reference
>60 years	23/570 (4.0)	27/241 (11.2)		50/811 (6.2, 4.6–8.0)	**<0.001**
**Etiology of primary adrenal insufficiency**					
Autoimmune	50/678 (7.4)	88/133 (66.2)	**<0.001**	138/811 (17.0, 14.5–20.0)	Reference
AIDS	304/678 (44.8)	5/133 (3.8)	**<0.001**	309/811 (38.1, 34.7–41.5)	**<0.001**
Tuberculosis	288/678 (42.5)	8/133 (6.0)	**<0.001**	296/811 (36.5, 33.2–39.9)	**<0.001**
ALD & Genetics	5/678 (0.7)	16/133 (12.0)	**<0.001**	21/811 (2.6, 1.6–3.9)	**<0.001**
Malignancy	27/678 (4.0)	7/133 (5.3)	0.500	34/811 (4.2, 2.9–5.8)	**<0.001**
Other	4/678 (0.6)	9/133 (6.8)	**<0.001**	13/811 (1.6, 0.8–2.7)	**<0.001**
**Associated autoimmune conditions**:					
Hypothyroidism	64/192 (33.3)	81/190 (42.6)	0.061	145/382 (38.0, 33.1–43.0)	Reference
Type 1 diabetes	60/192 (31.2)	51/190 (26.8)	0.343	111/382 (29.1, 24.6–33.9)	**0.009**
Graves’ disease	13/192 (6.8)	9/190 (4.7)	0.394	22/382 (5.8, 3.6–8.6)	**<0.001**
Pernicious anaemia	35/192 (18.2)	25/190 (13.2)	0.173	60/382 (15.7, 12.2–19.8)	**<0.001**
Premature ovarian	20/192 (10.4)	24/190 (12.6)	0.498	44/382 (11.5, 8.5–15.2)	**<0.001**

Results are presented as frequency (percentage) = N(%).

AIDS, acquired immunodeficiency syndrome; ALD, Adrenoleukodystrophy; CI, Confidence interval.

*Prevalence of primary adrenal insufficiency calculated out of total number of patients with adrenal insufficiency (N = 1180 for Public sector and N = 632 for Private sector).

^a^*P*-value: Comparison between Public and Private sectors;

^b^*P*-value: Comparison across the entire group of all patients with primary adrenal insufficiency.

n/N(%) = number of responses out of the total number of answers received.

Most patients, 442/811 (55%), presented with a constellation of the typical symptoms such as anorexia, nausea, vomiting, abdominal pain, and diarrhea in varying combinations. However, 126/811 (15.5%) presented in an adrenal crisis, as suspected by the practitioners [72/570 (12.6%) vs. 54/241 (22.4%); *p*<0.001] in public vs. private, respectively (Table 3).

**Table 3:** Reported presenting features and severity of the patients with adrenal insufficiency in the whole study group and by practice type.

Presenting features	Public & Academic	Private	*P*-value^a^	Total (%, 95% CI)	*P-*value^b^
**Gastrointestinal symptoms**	45/59 (76.3)	79/103 (76.7)	0.951	124/162 (76.5, 69.3–82.5)	
Nausea	35/49 (71.4)	66/87 (75.9)	0.570	101/136 (74.3, 66.9–81.6)	Reference
Vomiting	31/49 (63.3)	58/86 (67.4)	0.622	89/135 (65.9, 57.9–73.9)	0.134
Diarrhoea	30/49 (61.2)	46/80 (57.5)	0.676	76/129 (58.9, 50.4–67.4)	**0.008**
Abdominal pain	38/49 (77.6)	58/84 (69.0)	0.291	96/133 (72.2, 64.6–79.8)	0.700
Anorexia	30/49 (61.2)	50/82 (61.0)	0.977	80/131 (61.1, 52.7–69.4)	**0.021**
**Non-specific features**					
Dizziness	38/49 (77.6)	67/87 (77.0)	0.943	105/136 (77.2, 70.2–84.2)	Reference
Weight loss	37/52 (71.2)	64/86 (74.4)	0.675	101/138 (73.2, 65.8–80.6)	0.441
Salt craving	20/49 (40.8)	45/81 (55.6)	0.103	65/130 (50.0, 41.4–58.6)	**<0.001**
Skin pigment	28/49 (57.1)	56/86 (65.1)	0.358	84/135 (62.2, 54.0–70.4)	**0.007**
Backache	14/47 (29.8)	41/82 (50.0)	**0.025**	55/129 (42.6, 34.1–51.2)	**<0.001**
**The number of patients presenting with Adrenal crisis in 5yrs**:	72/570 (12.6)	54/241 (22.4)	**<0.001**	126/811 (15.5, 13.1–18.2)	
**Adrenal features suggestive of a crisis**:					
Hypoglycaemia	40/51 (78.4)	42/86 (48.8)	**0.001**	82/137 (59.8, 51.6–68.1)	Reference
Loss of conscious	25/48 (52.1)	27/82 (32.9)	**0.031**	52/130 (40.0, 31.6–48.4)	**0.001**
Shock	36/53 (67.9)	31/84 (36.9)	**<0.001**	67/137 (48.9, 40.5–57.3)	0.069

*Percentages of common presenting features according to respondent doctors (N = 59 for Public sector and N = 103 for Private sector) with non-responders censored for each question.

^a^*P*-value: Comparison between Public and Private sectors;

^b^*P*-value: Comparison across the entire group of all patients with primary adrenal insufficiency.

n/N(%) = number of responses out of the total number of answers received.

### Diagnostic strategies

In both public and private healthcare settings, the respondents used non-uniform methods to diagnose PAI, with a heavier reliance on clinical grounds and basic biochemistry to make the diagnosis in public, compared to the private healthcare setting (Table 4). In the public vs private setting, clinical grounds only were used in [24/39 (65.5%) vs 27/78 (34.6%); *p* = 0.006], clinical and serum sodium and potassium [32/44 (72.7%) vs 47/78 (60.3%); *p* = 0.166], clinical plus serum sodium, potassium plus cortisol [24/45 (53.3%) vs 49/78 (62.8%); *p* = 0.302], and finally by the combination of clinical criteria, electrolytes, low serum cortisol plus serum adrenocorticotropic hormone (ACTH) [42/52 (80.8%) vs 67/84 (79.8%); *p* = 0.886]. Despite the synthetic ACTH test (Synacthen^®^) being the standard special investigation to diagnose PAI, it was not universally available in public versus private healthcare settings. As a result, the proportions of practitioners who used clinical grounds alone versus a combination of clinical grounds together with electrolytes, serum cortisol, and serum or synthetic ACTH across the entire cohort were [51/117 (43.6%) vs. 109/136 (80.2%); *p*<0.001], respectively.

**Table 4:** Trends in diagnostic approaches practiced in the public and private sectors of health-care and availability of the diagnostic resources perceived by physicians practicing as a whole and in the two sectors.

Diagnostic approaches	Public & Academic (N = 59)	Private (N = 103)	*P-*value^1^	Total (%, 95% CI)	*P-*value^2^
**I. Basis of diagnosis** ^b^					
**a. Clinical features alone**					
• Never	15/39 (38.5)	51/78 (65.4)	**0.006**	66/117 (56.4, 47.4–65.4)	0.050
• Sometimes/often/always	24/39 (65.5)	27/78 (34.6)		51/117 (43.6, 34.6–52.6)	
**b. Clinical features plus serum sodium and potassium**					
• Never	12/44 (27.3)	31/78 (39.7)	0.166	43/122 (35.2, 26.8–43.7)	**<0.001**
• Sometimes/often/always	32/44 (72.7)	47/78 (60.3)		79/122 (64.8, 56.3–73.2)	**0.010**^c^
**c. Clinical features plus serum sodium, potassium plus cortisol**					
• Never	21/45 (46.7)	29/78 (37.2)	0.302	50/123 (40.6, 32.0–49.3)	**0.003**
• Sometimes/often/always	24/45 (53.3)	49/78 (62.8)		73/123 (59.4, 50.7–68.0)	**0.015**^c^
**d. Clinical features plus serum sodium, potassium, cortisol plus ACTH test**					
• Never	10/52 (19.2)	17/84 (20.2)	0.886	27/136 (19.8, 13.1–26.6)	**<0.001**
• Sometimes/often/always	42/52 (80.8)	67/84 (79.8)		109/136 (80.2, 73.4–86.8)	**<0.001**^c^
**II. Diagnostic test non-availability** ^a^^,^^b^					
**a. Basic diagnostic tests**					
• Never unavailable	14/48 (29.2)	51/81 (63.0)	**<0.001**	65/129 (50.4, 41.8–59.0)	0.383
• Sometimes/often unavailable	32/48 (66.7)	26/81 (32.1)		58/129 (45.0, 36.4–53.5)	
**b. Adrenal antibody**					
• Never unavailable	6/47 (12.8)	39/80 (48.8)	**<0.001**	45/127 (35.4, 27.1–43.8)	**0.031**
• Sometimes/often unavailable	32/47 (68.1)	30/80 (37.5)		62/127 (48.8, 40.1–57.5)	0.536^d^
**c. Serum cortisol**					
• Never unavailable	24/47 (51.1)	64/82 (78.1)	**0.006**	88/129 (68.2, 60.2–76.3)	**<0.001**
• Sometimes/often unavailable	19/47 (40.4)	15/82 (18.3)		34/129 (26.4, 18.8–34.0)	**0.002**^d^
**d. Serum ACTH**					
• Never unavailable	19/48 (39.6)	62/82 (75.6)	**<0.001**	81/130 (62.3, 54.0–70.6)	**<0.001**
• Sometimes/often unavailable	25/48 (52.1)	16/82 (19.5)		41/130 (31.5, 23.6–39.5)	**0.026**^d^
**e. Adrenal CT scan**					
• Never unavailable	18/48 (37.5)	54/81 (66.7)	**0.005**	72/129 (55.8, 47.2–64.4)	**0.002**
• Sometimes/often unavailable	25/48 (52.1)	22/81 (27.2)		47/129 (36.4, 28.1–44.7)	0.163^d^

Results are shown as frequency (percentage) = N(%).

ACTH, adrenocorticotropic hormone; CI, Confidence interval.

^a^”Often” and “very often” response combined as “often”.

^b^Percentages calculated out of total response given including “not sure” responses.

^c^Positive response (“Sometimes/often/always”) compared with positive response for use of “Clinical features” as a basis for diagnosis.

^d^Positive response (“Sometimes/often/always”) compared with positive response for non-availability of “Diagnostic tests”.

^1^*P*-value: Comparison between Public and Private sectors;

^2^*P*-value: Comparison across the entire group of all patients with primary adrenal insufficiency.

n/N(%) = number of responses out of the total number of answers received.

### Management patterns and barriers to optimal care

A greater variety of replacement therapy was available in the private, compared with the public healthcare systems, [98/570 (17.2%) vs. 106/241 (44.0%); *p*<0.001] of the patients were receiving hydrocortisone, whereas [95/570 (16.7%) vs. 108/241 (44.8%); *p*<0.001] were using fludrocortisone (Table 5). The remainder were replaced using, in decreasing frequency, prednisone [104/570 (18.2%) vs 81/241 (33.6%); *p*<0.001], cortisone acetate [11/570 (1.9%) vs 35/241 (14.5%); *p*<0.001], dexamethasone [15/570 (2.6%) vs 20/241 (8.3%) p<0.001] and betamethasone [0 (0.0%) vs 8/241 (3.3%); *p*<0.001]. The vast majority of patients 389/583 (66.7%) in the previous five years were not using any form of identification, indicating that they have PAI and need additional steroids in an emergency (Table 6). Our questionnaire attempted to elicit the barriers to optimal care for patients with PAI, shown in (Table 6). Areas that appeared to limit optimal care included lack of experience in managing an adrenal crisis and poor patient utilization of a form of medical identification indicating that they need steroids in emergency cases. Moreover, patient education, patients’ languages, and patient cultural issues appeared to undermine optimal healthcare delivery.

**Table 5:** Replacement management trends including drugs used, ways of doses adjustments and perceived drugs availability perceived by physicians in the whole study and in the public/academic and private sectors.

Management of PAI	Public/Academic (N = 570)	Private (N = 241)	*P*-value^1^	Total (%, 95% CI)	*P-*value^2^
**1. Therapeutic drugs usage**:					
Hydrocortisone	98/570 (17.2)	106/241 (44.0)	**<0.001**	204/811 (25.2, 22.2–28.2)	Reference
Fludrocortisone	95/570 (16.7)	108/241 (44.8)	**<0.001**	203/811 (25.0, 22.0–28.0)	0.926
Cortisone acetate	11/570 (1.9)	35/241 (14.5)	**<0.001**	46/811 (5.7, 4.1–7.3)	**<0.001**
Prednisone	104/570 (18.2)	81/241 (33.6)	**<0.001**	185/811 (22.8, 19.9–25.7)	0.258
Dexamethasone	15/570 (2.6)	20/241 (8.3)	**<0.001**	35/811 (4.3, 2.9–5.7)	**<0.001**
Betamethasone	0	8/241 (3.3)	**< 0.001**	8/811 (0.99, 0.3–1.6)	**<0.001**
**2. Basis of dose adjustment**^a^					
Body weight	31/48 (64.6)	51/84 (60.7)	0.659	82/132 (62.1, 53.8–70.4)	Reference
Body surface area	3/48 (6.2)	7/84 (8.3)	0.747	10/132 (7.6, 3.1–12.1)	**<0.001**
None (Fixed dose)	14/48 (29.2)	26/84 (31.0)	0.830	40/132 (30.3, 22.2–37.8)	**<0.001**
**3. Reported drug non-availability** ^a^^,^^b^					
Medicine					
Never unavailable	15/48 (31.2)	45/82 (54.9)	**0.015**	60/132 (46.2, 37.6–54.7)	0.901
Sometimes/often unavailable	26/48 (54.2)	33/82 (40.2)		59/132 (45.4, 36.8–53.9)	
Hydrocortisone					
Never unavailable	28/47 (59.6)	57/82 (69.5)	0.467	85/129 (65.9, 57.7–74.1)	**<0.001**
Sometimes/often unavailable	15/47 (31.9)	19/82 (23.2)		34/129 (26.4, 18.8–34.0)	
Fludrocortisone					
Never unavailable	13/47 (27.7)	30/84 (35.7)	0.623	43/131 (32.8, 24.8–40.9)	**<0.001**
Sometimes/often unavailable	27/47 (57.4)	44/84 (52.4)		71/131 (54.2, 45.7–62.7)	**<0.001**^c^
Prednisone					
Never unavailable	41/47 (87.2)	71/82 (86.6)	0.782	112/129 (86.8, 81.0–92.7)	**<0.001**
Sometimes/often unavailable	2/47 (4.26)	6/82 (7.32)		8/129 (6.2, 2.0–10.4)	**<0.001**^c^
Dexamethasone					
Never unavailable	18/47 (38.3)	47/84 (56.0)	0.095	65/131 (49.6, 41.0–58.2)	0.136
Sometimes/often unavailable	25/47 (53.2)	28/84 (33.3)		53/131 (40.5, 32.0–48.9)	**0.016**^c^
Betamethasone					
Never unavailable	20/46 (43.5)	53/83 (63.9)	0.067	73/129 (56.6, 48.0–65.1)	**<0.001**
Sometimes/often unavailable	16/46 (34.8)	16/83 (19.3)		32/129 (24.8, 17.4–32.2)	0.775^c^

Results are shown as frequency (percentage) = N(%).

CI, Confidence interval.

^a^Percentages according to respondent doctors (n = 59 for Public sector and 103 for Private sector).

^b^Percentages calculated out of total responses given including “not sure” responses.

^c^ Positive response (“Sometimes/often”) compared with positive response for non-availability of hydrocortisone.

^1^*P*-value: Comparison between Public and Private sectors;

^2^*P*-value: Comparison across the entire group of all patients with primary adrenal insufficiency.

n/N(%) = number of responses out of the total number of answers received.

**Table 6:** Some professionals and patients’-related barriers to optimal care perceived by physicians in the whole study and in the public/academic and private sectors.

Physician’s perspective on management of PAI	Public & Academic (N = 570)	Private (N = 241)	*P-*value^1^	Total (%, 95% CI)	*P-*value^2^
**1. Reported physicians’ experience in managing adrenal crisis**					
No patients	28/59 (47.5)	69/103 (67.0)	**0.015**	97/162 (59.9, 51.9–67.5)	Reference
1 patient	18/59 (30.5)	28/103 (27.2)	0.652	46/162 (28.4, 21.6–36.0)	**<0.001**
2–5 patients	11/59 (18.6)	5/103 (4.8)	**0.011**	16/162 (9.9, 5.8–15.5)	**<0.001**
> 5 patients	2/59 (3.39)	1/103 (0.97)	0.300	3/162 (1.8, 0.4–5.3)	**<0.001**
**2. Methods of patients’ identification**					
None	337/416 (81.0)	52/167 (31.1)	**<0.001**	389/583 (66.7, 62.7–70.5)	Reference
Other	11/416 (83.6)	4/167 (33.5)	1.00	15/583 (2.6, 1.4–4.2)	**<0.001**
Bracelet	54/416 (13.0)	87/167 (52.1)	**<0.001**	141/583 (24.2, 20.8–27.9)	**<0.001**
Card	14/416 (3.4)	24/167 (14.4)	**<0.001**	38/583 (6.5, 4.6–8.8)	**<0.001**
**3. Other recognized barriers to management**^a^					
Educational level	26/49 (53.1)	30/80 (37.5)	0.052	56/129 (43.4, 34.8–52.0)	Reference
Language issues	25/50 (50.0)	22/80 (27.5)	**0.013**	47/130 (36.1, 27.9–44.4)	0.232
Cultural issues	29/48 (60.4)	24/80 (30)	**0.001**	53/128 (41.4, 32.9–49.9)	0.745

Results are shown as frequency (percentage) = N(%).

CI, Confidence interval.

^a^Percentages according to respondent doctors (N = 59 for Public sector and N = 103 for Private sector).

^b^Percentages calculated out of total responses given including “not sure” responses.

^c^ Positive response (“Sometimes/often”) compared with positive response for non-availability of hydrocortisone.

^1^*P*-value: Comparison between Public and Private sectors;

^2^*P*-value: Comparison across the entire group of all patients with primary adrenal insufficiency.

n/N(%) = number of responses out of the total number of answers received.

## Discussion

Our survey suggests a PAI prevalence of 14.2 per million, which is considerably higher than previously determined 3.1 per million [16]. Our study also reveals that HIV, AIDS, and TB-related PAI are considerably more common than previously thought, with a higher likelihood of these conditions occurring in the more impoverished public healthcare sector than the more affluent private healthcare sector. We note with great concern a reliance on basic biochemistry, rather than definitive investigations to make the diagnosis and to establish the underlying etiology of PAI in South Africa. The replacement was predominantly determined by body weight, and there was an overall under-utilization of fludrocortisone, which is a significant concern.

It is interesting to note that even though this prevalence is higher than our previous case study, it is still significantly lower than the 140 percent observed in Europe [4]. Possible explanations for this significantly increased suggested prevalence could be the increased burden of HIV and TB, coupled with a growing awareness of PAI in South Africa. On the other hand, it is well known that the higher prevalence in Europe is predetermined by autoimmunity, the most important etiology of PAI in Europe [17]. We speculate that the low prevalence of autoimmunity among most of our black population in South Africa [10] could explain a similarly lower prevalence in South Africa. The shortage of endocrinologists and the low overall index of suspicion for PAI is the most plausible explanation for apparently low prevalence. Our public healthcare system is overwhelmed and under-resourced, limiting the widespread availability of Synacthen^®^ tests, autoantibody, and radiology investigations to make accurate diagnoses of PAI and assessment of their underlying etiology. Moreover, the Synacthen^®^ test is not readily available in South Africa, and as a non-registered drug, it needs to be imported with special dispensation from the Medicines Control Council: https://www.sahpra.org.za.

There was a preponderance of private, compared with government-employed practitioners, who managed patients with PAI. Across the grades, there were significantly more senior practitioners than the middle and junior ones. There were significantly more non-endocrine practitioners treating PAI in the state than in private, but no significant difference in the proportion of endocrinologists in both settings. Approximately (84%) of respondents practiced in urban areas, compared with rural areas. The majority of the patients, 70.3%, received care in public versus 29.7% in the private setting, yet the proportions of the South African population served by the public and private healthcare sectors are 85% and 15%. This implies that medical practitioners from the private healthcare sector, relative to its size, made PAI testing more readily available.

Autoimmunity predominated in the private healthcare system. In our survey, overall, there was a substantial contribution of HIV-related PAI in 33.6% and tuberculosis 32.2% to the entire cohort, but significantly TB and HIV dominated the public healthcare setting. The plausible explanation lies in the relative burden that the public shoulders with HIV and TB, compared to the private healthcare systems [15]. The SA population is overwhelmed by two simultaneously occurring epidemics of HIV and TB and a significant burden of non-communicable diseases [18,19]. The substantial contributions of HIV and TB in this survey warrant confirmation in a prospective prevalence study.

Autoimmune PAI contributed only 17% in this cohort, significantly less than our previously reported 50% [10]. However, when our analysis was stratified by poorly resourced public versus an affluent private healthcare system, many patients with autoimmunity were seen in the better-resourced private healthcare system. The likely explanation is that autoimmunity predominates in persons of European descent [3,10], whereas black Africans, due to substantial past inequity and deprivation, are more likely to frequent the public healthcare system. The true proportion of autoimmunity could not be determined due to the lack of available antibodies, particularly in the public sector. The basis for which autoimmunity was determined was based mainly on the physicians’ impression of an autoimmune etiology. The prevalence of coexistent type 1 diabetes and primary ovarian failure appear to be inflated in this cohort and are at odds with Western countries. It is speculated that recollection bias may have contributed to coexistent type I diabetes occurring in 29.1% of PAI.

Although there was no difference in the array of presenting symptoms in patients with PAI in public, compared with the private healthcare systems, a significant number presented with nausea, vomiting, anorexia, and diarrhea, which is similar to most studies [6], a high index of suspicion for PAI should be maintained globally. Particularly with the diagnoses of TB and HIV, as these may be distracting away from PAI, with potentially severe consequences [2]. South Africa has a poorly developed primary healthcare system, and it is expected that patients in our setting present late. Ross et al and Soule indicated that a substantial proportion of patients present either acutely or in a crisis [3,9].

It is interesting that although the adrenal crisis was reported in 15.5% across the entire cohort, features suggestive of a crisis such as hypoglycemia, shock, and loss of consciousness were reported as occurring more commonly among public healthcare practitioners, which is at odds with the reported numbers of adrenal crises in the private sector. This is likely due to the under-appreciation of the features of an adrenal crisis. If these features were correctly construed as compatible with an adrenal crisis, it is possible that there was a greater likelihood of adrenal crisis presenting to the public, compared with the private healthcare system. Another explanation for the under-reporting of adrenal crises maybe that non-specific symptoms were incorrectly attributable to AIDS. It was also intriguing to note that although the adrenal crisis was more prevalent among patients in the private sector, fewer respondents in the private sector had experience managing an adrenal crisis.

A significant proportion of patients who presented for the first time with PAI in an adrenal crisis usually resulted from a delayed diagnosis presented to the private healthcare system, despite a greater allocation of resources. We report an overall low utilization of medical alerts indicating that they need additional steroids in emergency cases, which may reveal a low disease literacy. This contrasts with the 89.9% utilization of a Medic Alert bracelet or necklace by participants in a 1997 North American survey [20].

Respondents from the private healthcare system had a wider variety of replacement glucocorticoid options from which to prescribe. A higher proportion of private doctors were using hydrocortisone and fludrocortisone as replacement therapy than their peers in the public setting. The possible explanation for the apparent limited use of these two treatment options could be the shortage of these drugs at local clinics where they are followed up. The limited use of fludrocortisone, which corrects for orthostatic hypotension, may also be associated with a limited number of endocrinologists involved in the survey. We remain concerned that the low utilization of mineralocorticoids in patients with PAI may be responsible for poor cardiometabolic outcomes and failure to restore well-being [21].

Treating physicians preferred weight-adjusted, rather than fixed-dose, or body surface area adjusted glucocorticoid doses. Weight-adjusted steroid dosing is the current recommended approach despite associated cardiac, bone, and other complications [22]. We speculate that the management’s finer nuances are not instituted where less specialized care for our patients exists. Elements in the management, such as minimizing overtreatment, cardiac complications, or under treatment with increased risk of crises in the event of major stressful episodes, are not universally appreciated [23,24].

Some of the reported barriers in diagnosing PAI in our study population were non-uniform availability of serum ACTH, auto-antibodies tests, and imaging facilities. As a result, several methods were relied upon to diagnose PAI, most importantly, clinical grounds and basic electrolytes, whereas the Synacthen^®^ stimulation test is not universally available. As South Africa has eleven official languages, significant language barriers could have played a role in suboptimal treatment of PAI in the public setting, more so than the private healthcare system.

Our study’s strengths include a broad spectrum of doctors who responded with significant experience of a rare medical condition. The survey has several weaknesses. It is presumed as this was an electronic questionnaire, it may be biased towards urban technically savvy clinicians rather than rural respondents because internet connectivity is predominantly limited to urban areas. We acknowledge that a survey methodology of this nature may pose severe weaknesses, particularly in the presence of missing responses, which could have introduced some degree of bias. Unfortunately, online surveys are limited by the quality and completeness and validity of the data sets [25]. Answer and selection bias may have influenced the numbers of patients. It is also conceivable that answers and selection biases may have led to prevalences inconsistent with the rest of the world data, representing an additional weakness.

Although recent data suggest mortality of PAI 2 to 3 times that of the general population, mortality and cardiovascular complications were not included in the survey [26]. Moreover, the Synacthen^®^ test’s availability was not determined by a direct question, thus representing critical omissions on our part. Questions relating to infections as a trigger for adrenal crises were not asked in the survey, which is a major weakness [26].

## Conclusions

We report the first survey of South African physicians’ perceptions of PAI, which suggests a considerably higher prevalence than previously reported. Patients presented with typical symptoms; however, 15.5% presented in adrenal crisis. There were significant disparities in respect of available diagnostic modalities and management options in the public versus the private settings. Greater awareness among health practitioners to timeously diagnose PAI is required to prevent a life-threatening outcome, especially in a country such as South Africa, where HIV and TB burdens are substantial.

## Supporting information

S1 Data Spreadsheet of all respondents and their responses. (XLSX)

S1 Appendix The survey questions with [potential responses] grouped under different domains (I, II and III). (DOCX)
