# Supplementary material for: Tiered healthcare in South Africa exposes deficiencies in management and more patients with infectious etiology of primary adrenal insufficiency
Source: PLoS One. 2020 Nov 5;15(11):e0241845. doi: 10.1371/journal.pone.0241845 (PMC7644036; doi:10.1371/journal.pone.0241845)
Supplement: S1 Appendix — (DOCX) [file pone.0241845.s002.docx]

| **Appendix I. The survey questions with [potential responses] grouped under different domains (I,II and III).** | |
| --- | --- |
| **I.** | **Respondents' profiles:** |
|  | 1. Please confirm that you are willing to participate in the survey [YES, I confirm that I am willing to participate in the study; No, I am not willing to participate in this survey.]* |
|  | 2. Where do you live currently? [Region (Africa, Middle East, Other) and country: ………..]** |
|  | 3. What is the best description that fits your professional status? [endocrinologist, nonendocrine specialist, GP]. |
|  | 4. Please tell us about your experience and professional grade (senior, middle grade, Junior). |
|  | 5. Please tell us about the type of your practice [university/government/private; hospital; clinic]. |
|  | 6. Please tell us about the locality of your practice [Urban/Rural]. |
| **II.** | **The clinical hypoadrenalism questionnaire:** |
|  | 1. Have you looked after patients with hypoadrenalism in your practice, in the last 5 years? [Yes/No]. |
|  | **2.** How many patients with hypoadrenalism have you looked after in your practice, in the last 5 years [please provide total numbers per gender: Males/Females]. |
|  | **3.** How many patients with Addison's disease have you had, in the past 5 years, excluding pituitary  cortisol deficiency, prior steroid use and patients with adrenalectomies? Indicate how many males and females in each age range?**.** ? [numbers per gender and age groups: 0-15 years; 16-30 years; 31-45 years; 46-60 years; 61-75 years; >75 years] |
|  | 4. How many patients with hypoadrenalism due to pituitary ACTH deficiency have you had in the past five years in your practice? [No …]. |
|  | 5. How many patients with hypoadrenalism due to prior steroid use have you had, in the past five years in your practice? [No … ]. |
|  | 6. How many patients with hypoadrenalism due to bilateral adrenalectomy have you had in the past 5 years in your practice? [No ….]. |
|  | 7. In your patients with Addison's disease, how many, in the past 5 years also have: 1. Type 1 diabetes mellitus; 2. Hypothyroidism; 3. Graves’ disease; 4. Pernicious anaemia; 5. Premature ovarian failure [No … ]. |
|  | 8. For the diagnosis of Addison's disease in the past 5years, Please indicate the frequency of the various bases of your diagnosis: [Options: 1. clinical ground only 2. clinical plus serum sodium (Na)/serum potassium (K) only 3. clinical plus serum sodium (Na)/serum potassium (K), plus antibodies 4. clinical plus low serum cortisol or synthetic ACTH stimulation test [Responses: Never/Sometimes/Often Very often/Invariably (always)]***. |
|  | 9. Which of the following clinical symptoms occurred in your patients with Addison's disease, in the past 5 years? [Options: self-reported increase in skin pigmentation/nausea/vomiting/weight loss/abdominal pain/backache/loss of consciousness/ Diarrhoea/salt craving/dizziness/shock/hypoglycaemia/anorexia; Responses: never/sometimes/often/very often/not sure]*** |
|  | 10. In the last 5 years, how many of your patients with Addison's disease presented with an adrenal (Addison's) crisis? [No]. |
|  | 11. For therapy: How many of your patients, in the past 5 years are on glucocorticoids (Cortisol/hydrocortisone) or mineralocorticoids to correct sodium and potassium? [HC …./FC ….]. |
|  | 12. In your patients with Addison's disease, how many, in the past 5 years are on the following therapy: [Options: Hydrocortisone/Cortisone acetate/Prednisone/Dexamethasone/Betamethasone]. |
|  | 13. How do you usually adjust the dose of glucocorticoids for your stable patients with Addison's disease? Options: Fixed dose for all patients/ dose adjusted for weight/dose adjusted for body surface area]. |
|  | 14. In your patients with Addison's disease, in the past 5 years, what do you think is the most likely cause? Indicate a number per diagnosis as (%) [Option: Autoimmune/ Addison's/ Adrenoleukodystrophy/ Tuberculosis/ AIDS-related/ malignancy/ Genetic/ Other/ Unknown]***. |
| **III.** | **Perceptions of Management of Addisons's Disease in Africa:** |
|  | 1. In your patients with Addison's disease, in the past 5 years, how many would you say it is easy, difficult or very difficult to control their symptoms? [Note that the total should be 100). |
|  | 2. How many of your patients, in the past 5 years use any form of identification indicating that they have Addison's disease and need steroids in case of emergency? (Note that the total should be 100): [medic alert bracelet/medic alert card/Other/none]. |
|  | 3. To what extent do agree/disagree with the given statements about the difficulties with diagnosing, managing and obtaining suitable treatment in your practice? [Options: Non-availability of dexamethasone; medicine; hydrocortisone; fludrocortisone; betamerhasone; prednisone ; diagnostic tests; serum Cortisol level; serum ACTH; adrenal antibody test and adrenal CT scan. Language issues/cultural issues/educational level; Responses: never/sometimes/often/very/often/not sure] [Responses: Never/ Sometimes/ Often/ Very often/ Not sure]. |
| * A logic is installed to allow only those consenting to proceed to the rest of the questionnaire. ** Only data derived from Africa are included in this report. *** On these matrixes: options are displayed as rows and responses are columns. | |
